# Effect of Polyphenols on Inflammation Induced by Membrane Vesicles from *Staphylococcus aureus*

**DOI:** 10.3390/cells13050387

**Published:** 2024-02-23

**Authors:** Yukino Oura, Yuko Shimamura, Toshiyuki Kan, Shuichi Masuda

**Affiliations:** 1 School of Food and Nutritional Sciences, University of Shizuoka, 52-1 Yada, Suruga-ku, Shizuoka 422-8526, Japanshimamura@u-shizuoka-ken.ac.jp (Y.S.); 2 Department of Synthetic Organic & Medicinal Chemistry, School of Pharmaceutical Sciences, University of Shizuoka, 52-1 Yada, Suruga-ku, Shizuoka 422-8526, Japan

**Keywords:** *Staphylococcus aureus*, membrane vesicles, inflammation, polyphenol

## Abstract

*Staphylococcus aureus*, a bacterium found on human skin, produces toxins and various virulence factors that can lead to skin infections such as atopic dermatitis. These toxins and virulence factors are carried in membrane vesicles (MVs), composed of the bacterium’s own cell membranes, and are expected to reach host target cells in a concentrated form, inducing inflammation. This study investigated the effects of two polyphenols, (–)-epigallocatechin gallate (EGCG) and nobiletin (NOL), on the expression of *S. aureus* virulence factors and the inflammation induced by MVs. The study found that EGCG alone decreased the production of Staphylococcal Enterotoxin A (SEA), while both EGCG and NOL reduced biofilm formation and the expression of virulence factor-related genes. When *S. aureus* was cultured in a broth supplemented with these polyphenols, the resulting MVs showed a reduction in SEA content and several cargo proteins. These MVs also exhibited decreased levels of inflammation-related gene expression in immortalized human keratinocytes. These results suggest that EGCG and NOL are expected to inhibit inflammation in the skin by altering the properties of MVs derived from *S. aureus*.

## 1. Introduction

*Staphylococcus aureus* is a prevalent bacterium on human skin, carried by approximately 5–30% of healthy individuals [1,2]. *S. aureus* produces various virulence factors, including Staphylococcal Enterotoxin A (SEA) and biofilms implicated in host cell attachment [3,4]. The accessory gene regulator (*agr*) quorum sensing system regulates the expression of *S. aureus* virulence factors [5]. RNAIII, an effector of *agr*, modulates the expression of downstream virulence factors like the hemolytic toxin beta-hemolysin gene (*hlb*) and the biofilm formation-regulating gene (*icaA*) [6]. Notably, SEA is produced early in proliferation and is not regulated by the quorum sensing system [7,8]. SEA interacts with keratinocytes, inducing cytokine production and causing skin inflammation [9,10].

*S. aureus* releases membrane vesicles (MVs), spherical vesicles approximately 20–200 nm in diameter, formed from bacterial membrane fragments [11]. *S. aureus*-derived MVs are produced not only after programmed cell death, but also throughout all growth stages and with different mechanisms. Although MV biogenesis and its mechanisms in Gram-positive strains are still largely unknown, several studies have shown that this process is tightly regulated and occurs in living cells [12]. MVs transport bacterial-derived molecules, including virulence factors, DNA, RNA, proteins, and bacterial signals, facilitating intercellular transport to host cells [13]. MVs, containing virulence factors such as SEA [13], adhere to and invade target host cells, potentially causing inflammatory reactions [10,14,15]. *S. aureus*-derived MVs have been reported to induce inflammatory cytokines in human keratinocytes, implicating their role in skin inflammatory diseases [16]. As MVs serve as carriers of bacterial-derived molecules to the host, controlling *S. aureus*-derived MVs is crucial to suppress inflammation.

**Figure 1:**
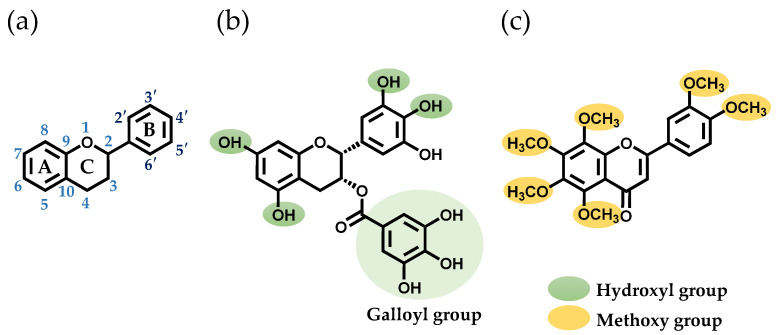
Structures of the polyphenols used in this study. (**a**) The basic skeleton and numbering system of a flavonoid. (**b**) The structure of (–)-epigallocatechin gallate (EGCG). (**c**) The structure of nobiletin (NOL).

In a prior study, researchers screened polyphenols that bind to SEA produced by *S. aureus* [17]. They discovered that two polyphenols, (–)-epigallocatechin gallate (EGCG) and nobiletin (NOL), altered the expression of genes related to SEA-induced inflammation in mouse spleen cells [18]. EGCG, a tea polyphenol, has been shown to have antioxidant, anti-inflammatory, and anticancer effects [19]. It was found that the interaction of SEA with the hydroxyl group at the 3″-position of the galloyl group of EGCG inhibits toxin activity [17]. NOL, a polyphenol found in citrus peels, has a flavone structure with multiple methoxy groups substituted into it. Like EGCG, NOL also has antioxidant, anti-inflammatory, and anticancer effects [19]. These polyphenols, which inhibit SEA-induced inflammation in *S. aureus*, may alter the virulence of MVs. The aim of this study is to investigate the effects of EGCG (Figure 1b) and NOL (Figure 1c) on the virulence of *S. aureus* and *S. aureus*-derived MVs, and the inflammation induced by MVs in keratinocytes.

The study compared the effects of EGCG and NOL on *S. aureus* growth, SEA production, biofilm formation, and the expression of genes related to virulence factors. MVs were prepared in broth containing EGCG and NOL, and their properties, including particle size, SEA content, and cargo protein, were examined. The study also considered the effect of MVs, whose properties had been altered in the presence of polyphenols, on the skin’s inflammatory response. Furthermore, the study evaluated the effect of MVs obtained in the presence of EGCG and NOL on the inflammatory response of keratinocytes.

## 2. Materials and Methods

### 2.1. Chemicals

A sample of SEA with a 95% purity level was acquired from Toxin Technology Inc., located in Sarasota, USA. The dilution process involved using phosphate-buffered saline (PBS) with a pH of 7.4, supplied by Thermo Fisher Scientific in Waltham, MA, USA. EGCG, sourced from Nagara Science Co., Ltd. in Gifu, Japan, was diluted to a concentration of 30 mM in 10% dimethyl sulfoxide (DMSO) obtained from FUJI-FILM Wako Pure Chemical Corporation (Osaka, Japan). Nobiletin, synthesized as per previous studies [20], was also diluted in 10% DMSO to a concentration of 30 mM. Each of these 30 mM polyphenol solutions was further diluted with MilliQ water.

### 2.2. Bacterial Strain and Culture Conditions

*S. aureus* C-29 strain, which produces Staphylococcal Enterotoxin A (SEA), was isolated from human hands [21]. The strain was introduced into brain heart infusion (BHI) broth from Oxoid Limited, Hampshire, UK, and subjected to incubation at 37 °C for 22 h with continuous shaking at 110 rpm. Following this, a 30 µL aliquot of the culture solution was transferred into 3 mL of BHI broth and further incubated at 37 °C for 18 h with shaking. After the incubation period, the culture solution was subjected to centrifugation at 11,600× *g* for 5 min, leading to the removal of the supernatant. The bacterial pellet underwent two washes with phosphate-buffered saline (PBS) and was then resuspended in PBS to generate a bacterial inoculum. To 0.9 mL of brain heart infusion (BHI) broth, 100 µL of the polyphenol solution was introduced, followed by the addition of 10 µL of the bacterial suspension. Subsequently, this mixture underwent further incubation at 37 °C for 24 h (or 4 to 5 h for gene expression analysis). The final concentrations of the polyphenols were as follows: 0.15, 0.3, 0.6, and 1.5 mM for EGCG, and 0.1, 0.3, 1.5, and 3.0 mM for NOL. The DMSO concentrations were 0.5%, 1.0%, 2.0%, and 5.0% for EGCG, and 0.3%, 1.0%, 5.0%, and 10% for NOL, respectively. The same DMSO concentration in each polyphenol solution was used as a control. The untreated solution was used as a negative control (0 mM), and the solution with a final concentration of 100 μg/mL kanamycin sulfate (FUJIFILM Wako Pure Chemicals Corporation) was used as a positive control (PC). The resulting culture solution was diluted with PBS, applied to mannitol salt agar, and subjected to incubation at 37 °C for 48 h, and subsequently the number of colonies was counted.

### 2.3. Measurement of Gene Expression in S. aureus

Following the incubation of the culture solution at 37 °C for 4 or 5 h, centrifugation at 11,600× *g* for 5 min was carried out, resulting in the removal of the supernatant and collection of the pellet. RNA extraction from the bacteria was performed using the RiboPureTM-Bacteria kit from Invitrogen, based in Carlsbad, CA, USA. The total RNA obtained was subsequently extracted, purified, and dissolved in RNase-free water following the kit’s protocol. The purity and concentration of the total RNA were assessed using a K2800 micro-spectrophotometer from Beijing Kaiao Technology Development Co., Ltd., Beijing, China. For further analysis, cDNA synthesis and real-time reverse transcription (RT)-PCR were conducted using the PrimeScript RT reagent kit from TaKaRa Bio Inc., located in Shiga, Japan. The targeted genes for analysis included the SEA gene (*sea*), the quorum sensing effector gene (RNAIII), the beta-hemolysin gene (*hlb*), and the biofilm formation-related gene (*icaA*) [22]. The 16S rRNA gene served as an internal standard to normalize mRNA levels among the test samples. The expression level of each gene was quantified relative to its expression level in the control.

### 2.4. Detection of SEA

The culture solution obtained after 24 h of incubation at 37 °C underwent centrifugation (11,600× *g*, 5 min), and the resulting supernatant was collected. Both the supernatant from the culture solution and isolated microvesicles (MVs) (diluted 5-fold with MilliQ water, with protein concentrations of 38.4 µg for control without EGCG and NOL, 35.4 µg for EGCG, and 36.6 µg for NOL) were then subjected to boiling for 5 min in a sample buffer. The sample buffer contained 1 M Tris-HCl (pH 6.8), 10% SDS, 0.5 mg/mL bromophenol blue, 25% 2-mercaptoethanol, and 20% glycerol. The proteins were separated by SDS-PAGE using a 15% polyacrylamide gel under a constant current of 20 mA for 90 min. SEA (at a final concentration of 100 ng/mL) was utilized as the loading control. Subsequently, the separated proteins were transferred from the gel to a PVDF membrane (Roche Molecular Biochemicals, Mannheim, Germany). Anti-rabbit SEA antibodies (Sigma-Aldrich, Saint Louis, MO, USA) and anti-rabbit alkaline phosphatase conjugate (KPL, Gaithersburg, MD, USA) served as the primary and secondary antibodies, respectively. The PVDF membrane was immersed in a BCIP/NBT solution (KPL) and incubated until color development occurred. Bands on the membrane were quantified using image analysis software (ImageJ 1.52a; National Institutes of Health, Bethesda, MA, USA). To clarify that a decrease in the number of bacteria did not lead to a decrease in SEA production, the amount of SEA protein in the culture supernatant was normalized by the number of cells.

### 2.5. Biofilm Formation of S. aureus

Tryptic soy broth (Difco, Detroit, MI, USA) containing 0.3 mM of each polyphenol and 10 μL of bacterial suspension was dispensed into a round-bottom 96-well plate, and subsequently incubated at 37 °C for 48 h. After aspirating planktonic cells, the biofilms were washed with sterile water. Subsequently, all the wells were augmented with 125 µL of 0.1% crystal violet solution (FUJIFILM Wako Pure Chemicals Corporation, Osaka, Japan). Following a 0 min incubation, any surplus crystal violet was eliminated, and the plates underwent three washes with sterile water before being left to air-dry. The crystal violet that had bound to the cells was dissolved in 150 µL of 100% ethanol obtained from FUJIFILM Wako Pure Chemicals Corporation. Biofilm formation was quantified by measuring absorbance at 595 nm using a SpectraMax 190 spectrophotometer (Molecular Devices Japan, Tokyo, Japan).

### 2.6. Isolation of MVs

A bacterial suspension (500 µL) was introduced into brain heart infusion (BHI) broth containing a 0.3 mM polyphenol solution (250 mL) and incubated at 37 °C for 17 h with shaking at 110 rpm. After incubation, the resulting culture solution underwent centrifugation (11,600× *g*, 5 min). The supernatant was then filtered through a 0.2 µm filter from Advantec Toyo Co., Ltd., Tokyo, Japan. The filtered culture supernatant was further processed by fractionation at 100 kDa using a centrifugal ultrafiltration filter unit (Amicon Ultra centrifugal filter devices 100 K; Merck Millipore, Darmstadt, Germany) employing the Himac CP-WX Series separation ultracentrifuge (Hitachi Koki Co., Ltd., Tokyo, Japan). The >100 kDa concentrate obtained was subjected to additional ultracentrifugation (150,000× *g*, 3 h) to isolate microvesicles (MVs) in the sediment fraction. The protein concentration of the isolated MVs was determined using a K2800 micro-spectrophotometer from Beijing Kaiao Technology Development Co., Ltd. The isolated MVs were stored at −20 °C until use, and unless specified otherwise, they were utilized in their undiluted form.

### 2.7. Particle Size Distribution of MVs

To 30 µL of microvesicles (MVs), PBS was added to achieve a total volume of 1 mL. The particle size distribution of *S. aureus*-derived MVs was then evaluated using dynamic light scattering (DSL) with a Zetasizer Ultra ZS Particle Analyzer from Malvern, UK. The Zetasizer software (ZS XPLORER 1.2.0.91) allows the reporting of intensity, volume, or number-based distributions. In this analysis, the particle size distribution of MVs was determined using number-based distributions, which typically represent the smallest size measured.

### 2.8. Identification of Cargo Proteins in MVs

The band patterns of cargo proteins in MVs were analyzed using SDS-PAGE. MVs were electrophoresed on 15% polyacrylamide gels and subsequently stained with Coomassie Brilliant Blue (CBB; Macalai Tesque, Inc., Kyoto, Japan) stain solution (50% methanol; Kanto kagaku, Tokyo, Japan, 7% acetic acid; Kanto kagaku, and 0.1% CBB) for 30 min. After staining, the gels were decolorized with a decolorizing solution (7% acetic acid; Kanto kagaku and 10% methanol; Kanto kagaku). Protein bands were excised from the gel, underwent trypsin digestion, and were subsequently analyzed through nano-LC/MS/MS. The procedures of protein digestion, nano-LC/MS/MS analysis, and mascot search were conducted by Japan Proteomics Co., Ltd. in Sendai, Japan. The quantification of bands on the gel was performed using image analysis software, specifically ImageJ 1.52a from the National Institutes of Health.

### 2.9. Inflammation-Related Gene Expression Induced by MVs

Human adult high-calcium low-temperature (HaCaT) cells (#300493; CLS Cell Lines Service, Eppelheim, Germany) [23] constitute an immortalized keratinocyte cell line established from the long-term primary culture of human adult skin keratinocytes under specific Ca^2+^ concentrations and temperature conditions. HaCaT cells were cultivated in MG-30 (DMEM, high glucose, ready-to-use, with serum, CLS Cell Lines Service), supplemented with penicillin-streptomycin (Sigma-Aldrich; penicillin (50 units/mL) and streptomycin (50 µg/mL)) at 37 °C, 5% CO_2_. In 96-well plates, HaCaT cells (4 × 10^4^ cells/well) were seeded and cultured for 24 h at 37 °C, 5% CO_2_. After the initial 24 h, each well received 10 µL of isolated microvesicles (MVs) (with or without polyphenols; protein concentration adjusted to 0.4 µg/µL) and was incubated for 6 h at 37 °C, 5% CO_2_. Dulbecco’s PBS (DPBS; Thermo Fisher Scientific) served as the control. A CellAmpTM Direct TB Green RT-qPCR Kit (TaKaRa Bio Inc.) was utilized for cell lysis, reverse transcription reactions, and real-time RT-PCR following the manufacturer’s protocol. The glyceraldehyde 3-phosphate dehydrogenase (GAPDH) gene was used as an internal standard to normalize mRNA levels among the test samples. The expression level of each gene is presented relative to the expression level in the control. The primer sequences used are presented in Table 1.

### 2.10. Statistical Analysis

The outcomes are presented as mean ± standard deviation (SD). Comparative analysis between two groups was conducted using a Student’s *t*-test. Differences among multiple groups were evaluated using one-way ANOVA with Dunnett’s multiple comparison post hoc test and one-way ANOVA with Tukey’s post hoc test. These statistical analyses were performed using Microsoft Excel 2019 (Microsoft, Redmond, WA, USA). A significance threshold of *p* < 0.05 was established for statistical significance.

## 3. Results

### 3.1. Effect of Polyphenols on Growth of S. aureus

**Figure 2:**
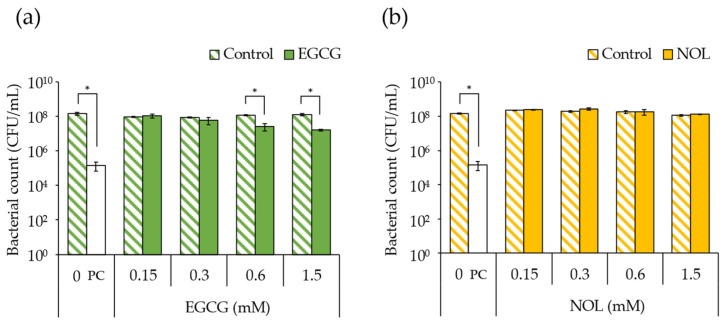
Impact of polyphenols on *Staphylococcus aureus* growth. (**a**) EGCG; (**b**) NOL. The concentrations used were as follows: 0.15, 0.3, 0.6, and 1.5 mM for EGCG, and 0.1, 0.3, 1.5, and 3.0 mM for NOL. The corresponding concentrations of dimethyl sulfoxide (control), in which each polyphenol was dissolved, were 0.5%, 1.0%, 2.0%, and 5.0% for EGCG, and 0.3%, 1.0%, 5.0%, and 10% for NOL. The untreated solution was used as a negative control (0 mM), and the solution with a final concentration of 100 μg/mL kanamycin was used as a positive control (PC). The relative bacterial count was determined based on the number of colonies on mannitol salt agar. The data are presented as the mean ± SD of three or more independent experiments. * indicates statistical significance (*p* < 0.05) compared to the control.

The impact of EGCG (final concentrations: 0.15, 0.3, 0.6, and 1.5 mM) and NOL (final concentrations: 0.1, 0.3, 1.5, and 3.0 mM) on the growth of *S. aureus* C-29 was investigated. The findings revealed that bacterial growth was inhibited, with a 4.6-fold decrease in 0.6 mM and a 7.8-fold decrease in 1.5 mM concentrations of EGCG (Figure 2a). In contrast, NOL did not exhibit inhibitory effects on bacterial growth (Figure 2b).

### 3.2. Effects of Polyphenols on Virulence Factor Gene Expression in S. aureus

**Figure 3:**
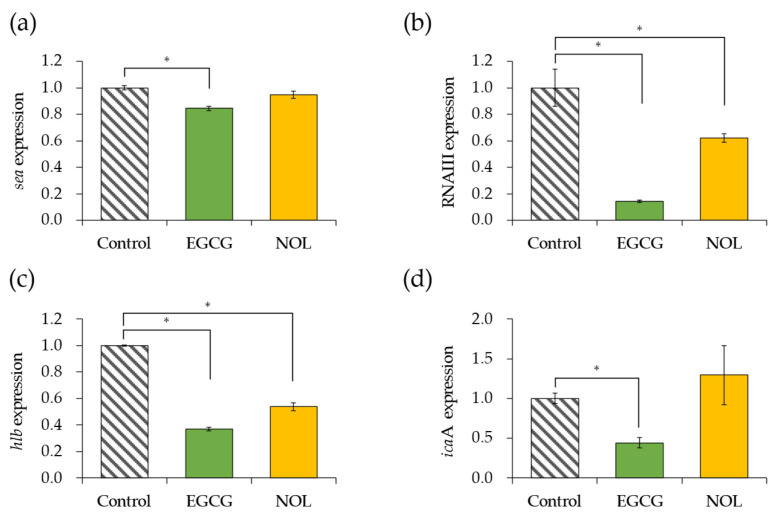
Impact of polyphenols on virulence factor gene expression in *Staphylococcus aureus*. (**a**) *sea*; (**b**) RNAIII; (**c**) *hlb*; (**d**) *icaA*. Each polyphenol (0.3 mM) was introduced to *S. aureus* and incubated for 4 or 5 h. As a control, 1% dimethyl sulfoxide dissolved in each polyphenol was used. The data are presented as the mean ± SD of three or more technical replicates for a single biological replicate each. * represents statistical significance (*p* < 0.05) compared to the control.

The impact of EGCG and NOL on the expression of four virulence factor genes in *S. aureus* was investigated. A concentration of 0.3 mM for each polyphenol was employed, ensuring that bacterial growth was not inhibited. Each gene was assessed at the specific incubation time (5 h for the *sea* and 4 h for the other genes) that it exhibited the highest expression in the control. As depicted in Figure 3a, EGCG led to a 1.2-fold decrease in *sea* (SEA gene) expression, while NOL did not induce any change. EGCG and NOL resulted in 7.0-fold and 1.6-fold decreases in the expression of RNAIII (the quorum sensing effector gene) (Figure 3b), and 2.7-fold and 1.9-fold decreases in the expression of *hlb* (the beta-hemolysin gene) (Figure 3c). Additionally, EGCG caused a 2.3-fold decrease in the expression of *icaA* (biofilm formation-related gene), whereas NOL did not affect it (Figure 3d).

### 3.3. Effect of Polyphenols on Biofilm Formation of S. aureus

**Figure 4:**
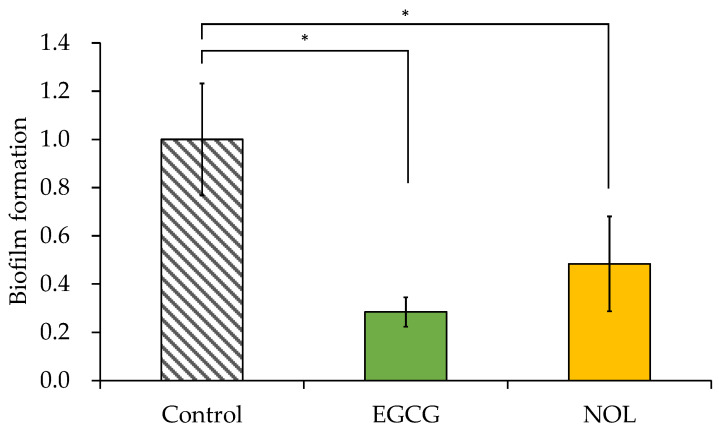
Effect of polyphenols on the biofilm formation of *Staphylococcus aureus*. Each polyphenol (0.3 mM) was introduced to *S. aureus* and incubated for 48 h. A 1% dimethyl sulfoxide solution, in which each polyphenol was dissolved, served as a control. The amount of biofilm formation was indicated relative to the absorbance at 595 nm in the control. The data are presented as the mean ± SD of three or more independent experiments. * represents statistical significance (*p* < 0.05) compared to the control.

The impacts of EGCG and NOL on biofilm formation in *S. aureus* were investigated using a concentration of 0.3 mM for each polyphenol that did not impede bacterial growth. As illustrated in Figure 4, EGCG and NOL exhibited a 3.5-fold reduction and a 2.1-fold reduction, with significant inhibitory effects on biofilm formation.

### 3.4. Effect of Polyphenols on Particle Size Distribution of S. aureus-Derived MVs

**Figure 5:**
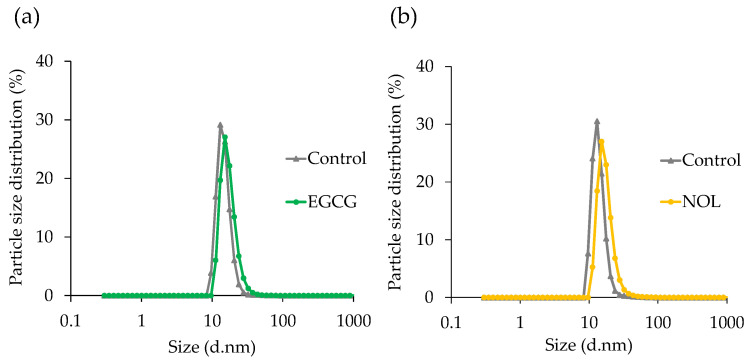
Effect of polyphenols on the particle size distribution of *Staphylococcus aureus*-derived membrane vesicles (MVs). (**a**) EGCG; (**b**) NOL. The dynamic light-scattering technique was employed to assess the average size distribution of MVs. The control consisted of 1% dimethyl sulfoxide instead of 0.3 mM polyphenols.

The particle size of MVs prepared with EGCG and NOL was determined using DLS. The mean diameter of each MV was 14.8 nm for the control without EGCG, 17.2 nm for EGCG treatment (Figure 5a), 14.0 nm for the control without NOL, and 17.5 nm for NOL treatment (Figure 5b). Both polyphenols had no discernible effect on the particle size distribution of *S. aureus*-derived MVs.

### 3.5. Effect of Polyphenols on SEA Production and SEA Content of MVs of S. aureus

**Figure 6:**
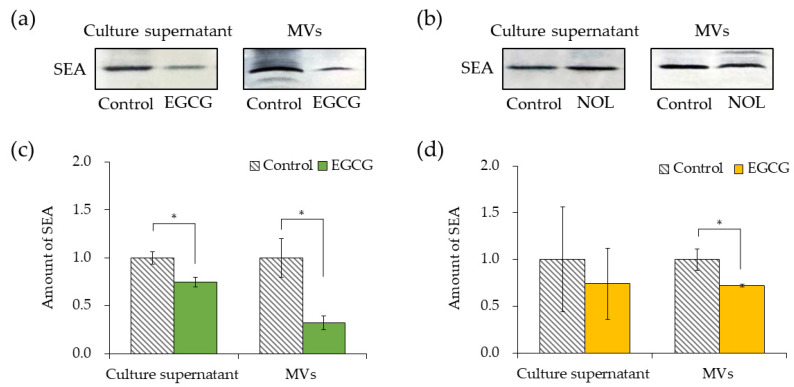
Comparison of the amount of SEA in the culture supernatant and membrane vesicles (MVs) derived from *Staphylococcus aureus* with polyphenols. (**a**) SEA production in the culture supernatant of *S. aureus* incubated with EGCG, and SEA content in *S. aureus*-derived MVs with EGCG (analyzed via SDS-PAGE and visualized through Western blotting). (**b**) SEA production in the culture supernatant of *S. aureus* incubated with NOL, and SEA content in *S. aureus*-derived MVs with NOL (analyzed via SDS-PAGE and visualized through Western blotting). (**c**) Quantification of SEA in the culture supernatant of *S. aureus* incubated with EGCG, and in MVs with EGCG. (**d**) Quantification of SEA in the culture supernatant of *S. aureus* incubated with NOL, and in MVs with NOL. Each MV sample underwent Western blot analysis using SEA antibodies; the same concentration of dimethyl sulfoxide (DMSO) in which each polyphenol was dissolved served as a control for the culture supernatant, and 1% dimethyl sulfoxide served as a control for polyphenol samples. Adobe Photoshop was employed for contrast and brightness adjustments to enhance visibility without altering the relative positions of polyphenol samples and controls. Data represent mean ± SD from three or more independent experiments. * denotes *p* < 0.05 compared to the control.

The impacts of EGCG (Figure 6a) and NOL (Figure 6b) on SEA production by *S. aureus* were investigated. A concentration of 0.3 mM for each polyphenol was employed, ensuring that bacterial growth was not hindered. The quantification of SEA produced per bacterium was determined by dividing the total amount of SEA produced by the number of bacteria. To clarify that a decrease in the number of bacteria did not lead to a decrease in SEA production, the SEA protein from the culture supernatant was normalized by the number of cells. EGCG demonstrated a 1.3-fold decrease in SEA production at 0.3 mM (Figure 6c). Conversely, NOL did not significantly affect SEA production (Figure 6d). The assessment of the impact of polyphenols (each at 0.3 mM) on SEA content in MVs revealed that they demonstrated a 3.1-fold and 1.4-fold decrease in SEA content in MVs prepared with EGCG (Figure 6c) and NOL (Figure 6d). Notably, while 0.3 mM NOL did not decrease SEA production (Figure 6d), the SEA content in MVs did not show a correlation with decreased SEA production.

### 3.6. Effect of Polyphenols on the Cargo Proteins in S. aureus-Derived MVs

**Figure 7:**
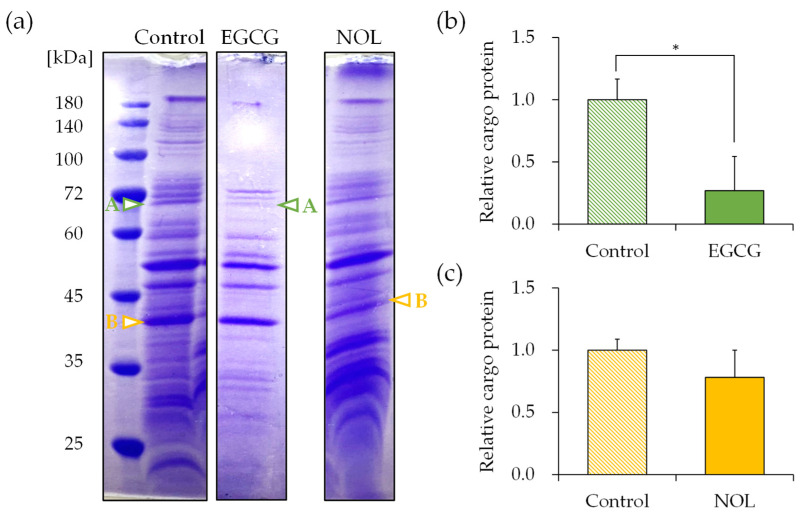
Effect of polyphenols on the cargo proteins in *Staphylococcus aureus*-derived membrane vesicles (MVs). (**a**) Cargo proteins in MVs with EGCG and NOL (1% dimethyl sulfoxide served as control). Adjustments in contrast and brightness were made using Adobe Photoshop for clarity without altering the relationship of polyphenol samples to control images. Arrows highlight downregulated protein bands. Bands A and B (indicated by arrows) underwent nano-LC–MS/MS analysis. (**b**) Quantification of decreased cargo protein band A in MVs with EGCG. (**c**) Quantification of decreased cargo protein band B in MVs with NOL. Data are presented as mean ± SD from three or more independent experiments. * indicates *p* < 0.05 compared to the control.

The protein concentrations of each MV were measured as 11.99 μg/μL for MVs prepared without polyphenols (control), 11.07 μg/μL for MVs prepared with EGCG, and 11.45 μg/μL for MVs prepared with NOL. SDS-PAGE was conducted to elucidate the effect of polyphenols on the cargo protein composition of MVs. To show the fold change for the proteins present in the MVs, the SDS-PAGE bands were quantified with ImageJ, as shown in figure below. The composition of MVs prepared with 0.3 mM of each polyphenol exhibited distinct changes. Subsequently, proteins in *S. aureus*-derived MVs whose expressions were reduced by polyphenols were identified using nano-LC-MS/MS analysis. Several significantly decreased protein bands were cut out for identification; proteins around 70 kDa (Figure 7a; band A) for EGCG (Figure 7b) and proteins around 40 kDa (Figure 7a; band B) for NOL (Figure 7c) in the MVs were listed because they showed a reliable score in the Mascot search engine (http://www.matrixscience.com, accessed on 12 January 2021). The protein bands around 70 kDa decreased by EGCG were identified as the dihydrolipoyllysine-residue acetyltransferase component of the pyruvate dehydrogenase complex, formate acetyltransferase, and chaperonin GroEL (Table S1a). The bands around 40 kDa decreased by NOL were identified as glyceraldehyde-3-phosphate dehydrogenase, alcohol dehydrogenase, and L-lactate dehydrogenase (Table S1b).

### 3.7. Effects of Polyphenols on Expression of Inflammation-Related Genes Induced by S. aureus-Derived MVs

**Figure 8:**
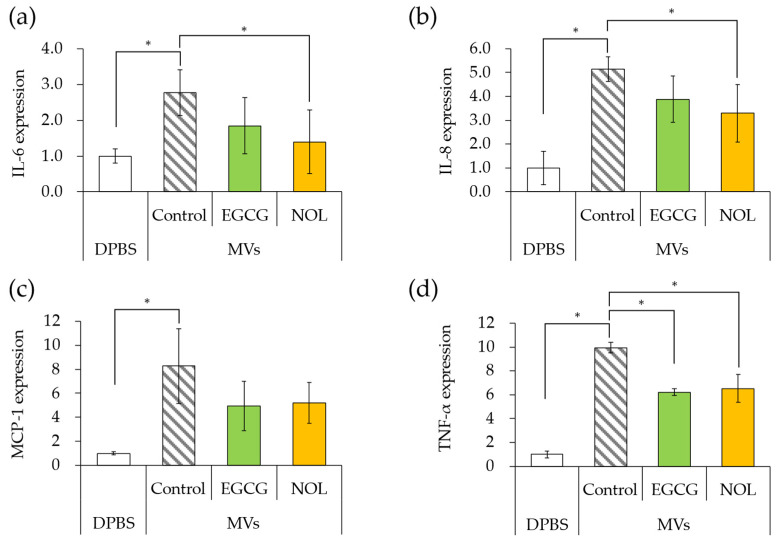
Expression levels of inflammation-related genes induced by membrane vesicles (MVs) prepared with polyphenols. (**a**) interleukin-6 (IL-6); (**b**) interleukin-8 (IL-8); (**c**) monocyte chemoattractant protein-1 (MCP-1); (**d**) tumor necrosis factor (TNF-α). MVs prepared with EGCG or NOL were introduced to human adult low-calcium high-temperature (HaCaT) cells to assess the expression of inflammation-related genes. Dulbecco’s phosphate buffer (DPBS) served as a control instead of MVs. Control MVs comprised 1% dimethyl sulfoxide without 0.3 mM polyphenols. Asterisks represent significant differences (Tukey–Kramer test, *p* < 0.05). Values represent the mean ± SD from three independent experiments.

The impact of MVs prepared with EGCG or NOL on the expression of inflammation-related genes in HaCaT cells was investigated. The amount of MVs added to the cells was adjusted to equal 20 μg/mL of protein concentration. The addition of MVs (without polyphenols) to HaCaT cells resulted in 2.8-fold, 5.1-fold, 8.3-fold, and 10-fold increases in the expression of IL-6 (Figure 8a), IL-8 (Figure 8b), MCP-1 (Figure 8c), and TNF-α (Figure 8d) compared to DPBS (without MVs). When MVs prepared with EGCG were added to HaCaT cells, the expression of TNF-α (Figure 8d) was significantly downregulated (1.6-fold decrease), and there was a tendency to downregulate IL-6 (Figure 8a), IL-8 (Figure 8b), and MCP-1 (Figure 8c) compared to the control MVs (without polyphenols) by 1.5-fold, 1.3-fold, and 2.5-fold. Similarly, when MVs prepared with NOL were added to HaCaT cells, the expressions of IL-6 (Figure 8a), IL-8 (Figure 8b), and TNF-α (Figure 8d) tended to downregulate (2.0-fold, 1.6-fold, and 1.5-fold), and MCP-1 (Figure 8c) was significantly downregulated (1.6-fold) compared to the control MVs (without NOL).

## 4. Discussion

This study investigated the effects of EGCG and NOL on both *S. aureus* and its MVs. Initially, the impact of polyphenols on *S. aureus* virulence was examined. EGCG inhibited *S. aureus* growth at a final concentration of 0.6 mM, while NOL did not exhibit growth inhibition even at 3.0 mM. EGCG has been shown to inhibit the growth of a wide range of Gram-positive bacteria [19]. On the other hand, the minimum inhibitory concentration (MIC) of EGCG against *S. aureus* was reported to be 0.2 mM, showing weak activity [24]. In this study, antibacterial activity was relatively weak, as growth was inhibited at 0.6 mM. This is because the growth inhibitory effect of EGCG was measured in BHI broth, so this may be due to the interaction of BHI broth-derived proteins with EGCG, resulting in a decrease in its activity. The hydroxy or galloyl groups of EGCG form hydrogen bonds with the bacterial peptidoglycan layer, leading to the disruption of cross-links in the peptidoglycan layer and bacterial degradation [25]. NOL, a polymethoxyflavone with a methoxy group replacing the hydroxy group, has an MIC of 4.0 mM against *S. aureus* [26]. Additionally, tangeretin, which has a methylated hydroxy group, also has an MIC of 4.3 mM against *S. aureus* [26], suggesting that the presence of a hydroxy group may contribute to antimicrobial activity. Moreover, the galloyl group has been reported to interact more strongly with cell membranes [27]. These findings suggest that EGCG exhibits greater antimicrobial activity than NOL when compared at the same concentration (0.3 mM). Given that MVs are formed from cell membranes, it is assumed that the alteration in membrane fluidity due to the interaction with polyphenols plays a crucial role in determining the properties of MVs.

The study then investigated the impact of polyphenols on the gene expression of virulence factors in *S. aureus*. EGCG demonstrated the suppression of *sea* expression, while NOL did not exhibit such suppression. SEA is implicated in inflammatory cytokine responses [8], implying that EGCG may have the capability to suppress inflammation induced by *S. aureus*. Both EGCG and NOL significantly suppressed the expression of RNAIII and the beta-hemolysin gene (*hlb*). While EGCG significantly downregulated the expression of *icaA*, NOL did not exhibit suppression. RNAIII plays a crucial role in regulating quorum sensing, a mechanism that controls the expression of various pathogenic factors [28]. Beta-hemolysin damages keratinocytes, contributing to its establishment in the skin [3]. *icaA* is involved in biofilm formation, enhancing resistance to antibiotics [29]. These findings suggest that both EGCG and NOL may mitigate inflammation by inhibiting the expression of virulence factors in *S. aureus*.

EGCG and NOL significantly inhibited biofilm formation in *S. aureus*. EGCG is believed to bind to the peptidoglycan layer [30] and interfere with the initial stages of biofilm formation, which relies on hydrophobic interactions between the bacterial cell wall and the surface where colonies form [27]. It is plausible that NOL, through a similar mechanism, disrupts the cell wall, leading to the inhibition of biofilm formation. Biofilm plays a crucial role in the survival of *S. aureus* in challenging environments [31]. Therefore, the addition of EGCG and NOL can aid in inhibiting the growth of *S. aureus* by impeding biofilm formation.

Considering that polyphenols were found to inhibit the virulence of *S. aureus*, their effects on MVs were investigated. The incubation time was set to 17 h, as previous studies indicated the stability of *S. aureus*-derived MVs at this duration [22]. The particle size of MVs prepared with EGCG and NOL was measured, revealing that each polyphenol had no effect on the particle size distribution of MVs. Although it was anticipated that polyphenols interacting with the cell membrane of *S. aureus* might alter membrane fluidity [32], these changes did not necessarily affect the particle dimension of MVs. As polyphenols were expected to modify the cargo proteins of MVs, their impact on the amount of SEA in MVs was assessed. EGCG and NOL significantly reduced the amount of SEA in MVs. While the amount of SEA production by *S. aureus* was suppressed by EGCG but not by NOL, the quantity of SEA produced did not correspond to the amount of SEA in the MVs. This finding aligns with previous studies [22] suggesting that many proteins encapsulated in *S. aureus* MVs are associated with the cell membrane, and the selection of components for encapsulation in MVs may occur [33]. These results imply that EGCG and NOL may inhibit the internalization of *S. aureus* virulence factors involved in inflammation into MVs.

The impact of polyphenols on the cargo proteins of *S. aureus*-derived MVs was examined, revealing several proteins reduced in MVs cultured in the presence of EGCG or NOL. Despite the polyphenols not affecting the particle size of MVs, this suggests that there is no relationship between the particle size of MVs and the composition of cargo proteins. Proteins around 70 kDa attenuated in *S. aureus*-derived MVs cultured with EGCG included chaperonin GroEL, known to be involved in bacterial growth [34]. The treatment of *S. aureus* with catechins has been reported to downregulate chaperone proteins like GroEL [35], supporting the correlation between secretome proteins of *S. aureus* and the cargo proteins of MVs [33]. Molecular chaperones play a role in maintaining stability by interacting with proteins to assist in their conformational formation and inhibit aggregation [34]. EGCG was suggested to reduce the molecular chaperone GroEL, leading to the decreased expression of several cargo proteins. Furthermore, several proteins involved in the glucose metabolism, such as L-lactate dehydrogenase, were included as proteins around 40 kDa attenuated in *S. aureus*-derived MVs cultured with NOL. The addition of glucose to the broth has been reported to promote *S*. *aureus* biofilm formation [36], and MVs themselves are considered to function as a matrix, as the backbone of biofilm [37]. The decrease in several proteins involved in the glucose metabolism in MVs suggested that NOL inhibits MV-mediated biofilm formation.

As polyphenols altered the cargo proteins of MVs, their effect on the induction of inflammation in HaCaT cells was examined. Previous studies demonstrated that MVs upregulated the expression of IL-6, IL-8, MCP-1, and TNF-α in HaCaT cells [22], making these inflammation-related genes the focus. *S. aureus*-derived MVs cultured with EGCG and NOL suppressed the expression of inflammation-related genes induced by MVs. SEA-producing *S. aureus* was reported to promote the expression of IL-6, IL-8, and TNF-α in HaCaT cells [38], and the addition of SEA to HaCaT cells promoted the expression of IL-6, IL-8, and MCP-1 [9]. These results suggest that polyphenols reduce SEA in MVs and suppress the expression of inflammation-related genes. Although MVs were corrected for the amount of protein, not for the number of MVs, differences in the inhibitory effect of polyphenols on the induction of inflammation by MVs may be due to differences in the number of MVs. Future research should focus on identifying the components involved in inflammation induction by *S. aureus*-derived MVs.

Catechins, including EGCG, target bacterial membrane proteins and fatty acid synthesis, leading to alterations in membrane fluidity in Gram-positive bacteria [39]. The mechanism involves catechins binding to lipid heads near the bilayer surface of the cell membrane, forming hydrogen bonds. The ratio of these bonds increases with the number of hydroxyl groups present in catechins. The hydrogen bonding disrupts cell membranes, contributing to the antimicrobial effects of catechins [40]. The hydroxylation patterns at C5, C7, C3, and C4′ of the flavonoid structure are crucial for the antibacterial activity of catechins [41]. Nobiletin, having a polymethoxylated structure, exhibits lower antimicrobial activity against several bacteria, including *S. aureus* [25]. On the other hand, in order for flavonoids to act on bacterial cell membranes, they must retain amphiphilic properties to penetrate bacteria. Flavonoids with high hydroxylation levels may face challenges in terms of lipophilicity, affecting their ability to cross pathogen cell membranes. On the other hand, lipophilic flavonoids like nobiletin can penetrate lipid bilayer membranes, inducing changes in membrane fluidity and accessibility [42]. Further studies are needed to elucidate the specific mechanisms by which structural features of EGCG and NOL affect MVs.

In this study, only one growth condition in the presence of EGCG and NOL was investigated. Phenol soluble modulin (PSM) peptides are a type of cytolytic peptide toxin promoting the release of *S. aureus*-derived MVs [43]. A large amount of PSM was present in the MVs prepared in this study after 17 h of incubation [22], which may be involved in the destruction of MVs. Thus, different results might be obtained at different stages of polyphenol addition since the properties of *S. aureus*-derived MVs differ depending on the growth stage. For future perspectives, changes in MV composition under different growth and stress conditions must be evaluated in more detail.

## 5. Conclusions

This study aimed to demonstrate the potential of polyphenols, specifically EGCG and NOL, to inhibit the inflammatory responses mediated by *S. aureus*-derived MVs. Our findings revealed that these polyphenols influence the inclusion of SEA and cargo proteins in *S. aureus*-derived MVs. Furthermore, we found that both EGCG and NOL inhibited inflammatory reactions by altering the inclusion components of MVs. In conclusion, this study provides new insights into the modulation of inflammation induced by *S. aureus*-derived MVs. It highlights the potential of polyphenols, particularly EGCG and NOL, as promising therapeutic agents for managing inflammation associated with *S. aureus.*

## Figures and Tables

**Table 1:** Primer sequences for gene expression analysis of HaCaT cells.

Primer	Forward Primer (5’ to 3’)	Reverse Primer (5’ to 3’)
GAPDH	GGACCTGACCTGCCGTCTAG	GAGGAGTGGGTGTCGCTGTT
IL-6	TGGCTGAAAAAGATGGATGCT	TCTGCACAGCTCTGGCTTGT
IL-8	TTGGCAGCCTTCCTGATTTC	TGGTCCACTCTCAATCACTCTCA
MCP-1	TCGCTCAGCCAGATGCAAT	TGGCCACAATGGTCTTGAAG
TNF-α	CCCAGGGACCTCTCTCTAATC	ATGGGCTACAGGCTTGTCACT

GAPDH was used as an internal standard.

## Data Availability

No new data were created or analyzed in this study. Data sharing is not applicable to this article.

## Supplementary Materials

The following supporting information can be downloaded at: https://www.mdpi.com/article/10.3390/cells13050387/s1, Table S1: Cargo proteins in MVs with downregulated expression levels by (a) EGCG and (b) NOL.
