# Supplementary material for: Effect of Polyphenols on Inflammation Induced by Membrane Vesicles from Staphylococcus aureus
Source: Cells. 2024 Feb 23;13(5):387. doi: 10.3390/cells13050387 (PMC10931263; doi:10.3390/cells13050387)
Supplement: Supplementary file 1 [file cells-13-00387-s001.zip › cells-2809097-supplementary.pdf]

Table S1. Cargo proteins in MVs with downregulated expression level by (a) EGCG or (b) NOL.

(a)

Biological Process and molecular function	Protein	gene	MW	Score	Coverage	Accession
Glycolytic process	Dihydrolipoyllysine-residue acetyltransferase-component of pyruvate dehydrogenase complex	*pdhC*	46,382	512	50	Q5HGY9
Glucose metabolic process	Formate acetyltransferase	*pflB*	84,862	257	21	Q2YV53
Protein refolding	Chaperonin GroEL	*groEL* *groL*	57,614	127	23	Q6GF43

(b)

Biological Process and molecular function	Protein	gene	MW	Score	Coverage	Accession
Arginine biosynthetic process	Ornithine carbamoyltransfelase	*arg*F	37,517	222	31	Q5HGR3
Glucose metabolic process, glycolytic process	Glyceraldehyde-3-phosphate dehydrogenase 1	*gapA*1 *gap*	36,281	187	31	Q5HHP5
Arginine biosynthetic process, arginine catabolic process to ornithine	Ornithine carbamoyltransferase, catabolic	*arcB*	37,731	126	17	Q6GDG8
Alcohol dehydrogenase (NAD^+) activity, zinc ion binding	Alcohol dehydrogenase	*adh*	36,048	123	16	Q2YSX0
Glycolytic process	L-lactate dehydrogenase 2	*ldh2*	34,420	88	15	Q2FDQ7
D-lactate dehydrogenase activity, NAD binding	D-lactate dehydrogenase	*ldhD* *ddh*	36,682	70	16	Q5HD29
Glycolytic process	Pyruvate dehydrogenase E1 component subunit- alpha	*pdhA*	41,383	44	5	Q5HGZ1
